# Joint External Evaluation scores and communicable disease deaths: An ecological study on the difference between epidemics and pandemics

**DOI:** 10.1371/journal.pgph.0000246

**Published:** 2022-08-11

**Authors:** Vageesh Jain, Ashley Sharp, Matthew Neilson, Daniel G. Bausch, Thomas Beaney

**Affiliations:** 1 Institute for Global Health, University College London (UCL), London, United Kingdom; 2 UK Public Health Rapid Support Team, UK Health Security Agency/London School of Hygiene and Tropical Medicine, London, United Kingdom; 3 Independent Public Health Consultant, London, United Kingdom; 4 FIND, The Global Alliance for Diagnostics, Geneva, Switzerland; 5 Imperial College London, London, United Kingdom; Universidad Nacional de Colombia, COLOMBIA

## Abstract

The Joint External Evaluation (JEE) assesses national capacities to implement the International Health Regulations (IHR). Previous studies have found that higher JEE scores are associated with fewer communicable disease deaths. But given the impact of COVID-19 in many countries, including those believed to have developed IHR capacities, the validity of the JEE for pandemic preparedness has been questioned. We constructed univariable and multivariable linear regression models to investigate the relationship between JEE scores and i) deaths from communicable diseases before the pandemic and ii) deaths from COVID-19. We adjusted for country differences in age, health system access, national wealth, health expenditure, democratic governance, government restrictions, pre-pandemic tourist arrivals and testing capacity (estimated by test positivity rates). For COVID-19 deaths, we calculated cumulative deaths per 100,000 at 3, 6 and 12 months into the pandemic. A total of 91 countries were included, with a median JEE score of 50%. On multivariable linear regression the association between JEE scores and log COVID-19 deaths was significant and positive at 3 months (β 0.05, p = 0.02), becoming statistically non-significant, at 6 (β 0.02, p = 0.27) and 12 months (β -0.03, p = 0.19), while the association with log communicable disease deaths was significant and negative (β -0.03, p = 0.003). A higher Stringency Index was significantly associated with higher log COVID-19 deaths at 3 (β 0.04, p = 0.003) and 6 (β 0.04, p = 0.001) months, but not at 12 months (β 0.02, p = 0.08). Higher test positivity rates were associated with higher log COVID-19 deaths at all time points, at least partially attenuating the positive association between Stringency Index and log COVID-19 deaths. While universal health coverage indices (β -0.04 p<0.001) and international tourist arrivals were associated with log communicable disease deaths (β 0.02, p = 0.002), they were not associated with log COVID-19 deaths. Although the same tool is used to assess capacities for both epidemics and pandemics, the JEE may be better suited to small outbreaks of known diseases, compared to pandemics of unknown pathogens.

## Introduction

The 2005 International Health Regulations (IHR) provide an overarching framework to assist countries to “prevent, protect against, control and provide a public health response to the international spread of disease” and defines countries’ rights and obligations in handling emergencies that have the potential to cross borders. The Joint External Evaluation (JEE) is a voluntary, collaborative, multisectoral process led by national governments and supported by the World Health Organization (WHO) that aims to assess and monitor national capacities to implement IHR, with a view to identifying opportunities to strengthen preparedness and response.

Disease outbreaks are often unpredictable and require a range of approaches for preparedness and response that may or may not be adequately addressed by IHR and JEE. The unprecedented scale of the COVID-19 pandemic contrasts greatly with the more typical and much more frequent local epidemics of known diseases, such as cholera and yellow fever [1]. Given the severe impact COVID-19 has had in nations largely believed to be operating in line with IHR, and thus prepared to confront outbreaks, the relevance of IHR competencies and validity of the JEE have been questioned. At the request of Member States, WHO recently convened a Review Committee on the Functioning of IHR during COVID-19, concluding that much of what is contained within the current IHR is appropriate and well-considered, but was not sufficiently implemented prior to the pandemic [2].

The Global Health Security Index (GHSI) was developed in 2019, as an independent adjunct to the JEE. A 2020 regression analysis concluded that, for both the JEE and the GHSI, higher total scores were associated with fewer deaths from communicable diseases in general [3]. However, a separate analysis showed that these scores were not correlated with COVID-19 mortality across countries during the early phase of the pandemic [4], although the study failed to account for important confounders. A later regression analysis showed that higher JEE scores were correlated with fewer COVID-19 deaths [5], but adjusted for testing rates (likely to reflect the true numbers of cases) as a proxy for testing capacity, with only a very limited set of countries in multivariable analysis.

To improve pandemic preparedness and risk assessment, it is necessary to understand the extent to which the relationship between national scores on the JEE and deaths from COVID-19 differs from that for other communicable diseases. This analysis will support policy makers to review the utility of the JEE in assessing preparedness against both epidemics and pandemics.

## Methods

### Investigated variables and sources

In this ecological study, we reviewed all published JEE reports available in the public domain on the WHO website [6] and obtained data on a range of other factors that may affect the relationship between JEE scores and deaths from COVID-19 or other communicable diseases. The Oxford COVID-19 Government Response Tracker Stringency Index combines nine different indicators: school closures, workplace closures, cancellation of public events, restrictions on public gatherings, closures of public transport, stay-at-home requirements, public information campaigns, restrictions on internal movements, and international travel controls [7]. We recorded the Stringency Index on a scale of 0 to 100 for each country at 2, 5 and 11 months into the pandemic, reflecting a time lag between restrictive government policies and any potential impact on COVID-19 deaths (which were measured at 3, 6 and 12 months). This assumed a median lag of 10 days between a change in government mobility restrictions and community rates of infection [8], five days from infection to symptom onset, and a further 16 days from symptom onset to possible death [9]. We obtained 2019 data on national population size, the proportion of the population aged ≥65, Gross National Income per capita, % Gross Domestic Product (GDP) spent on healthcare, and universal health coverage (UHC) service index (2017) from the World Bank public database [10]. We also collated data on pre-pandemic international tourist arrivals (as a proxy for international travel) from the UN World Tourism Organization Dashboard [11] and data on the strength of democratic governance through the Economist Intelligence Unit’s (EIU) 2020 democracy index. The EIU democracy index covers 5 domains, combining 60 indicators, and is measured on a scale of 0 to 100 from the least to the most democratic [12]. Higher scores have been associated with lower excess mortality rates due to the COVID-19 pandemic in high-income countries [13].

We included test positivity rate as a proxy for testing capacity and a potential confounder which could affect the relationship between JEE and deaths. The fraction of tests that return a positive result can provide an indication as to the adequacy of a COVID-19 testing programme and the reliability of death statistics; a low test positivity rate suggests low transmission and sufficient surveillance capacity, whereas a high test positivity rate suggests high transmission and inadequate testing, with many COVID-19 deaths undetected and unrecorded [14]. We combined routinely available data on COVID-19 tests [15] with data on cases [16] to calculate test positivity (cases per 100,000/tests per 100,000) across countries at 3, 6 and 12 months into the pandemic.

From 2016–18 most JEE reports were published in the same format, while later assessments included a slightly different scoring system. We extracted data in the same way but adjusted to ensure comparability across countries, converting all raw JEE scores to percentages representing the proportion of the maximum possible score obtained by a country.

### Outcome measures

The WHO declared the COVID-19 outbreak a pandemic on 11^th^ March 2020 [17]. We calculated cumulative COVID-19 deaths per 100,000 population for each country at 3 months (10^th^ June 2020), 6 months (10^th^ September 2020) and 12 months (10^th^ March 2021) into the pandemic, by dividing recorded COVID-19 deaths [18] by population size [19] and multiplying by 100,000. We used the Global Burden of Disease study [20] to similarly collate data on communicable disease deaths per 100,000 in 2019 for each country (i.e. prior to the COVID-19 pandemic), excluding deaths from maternal, neonatal and nutritional diseases.

### Data extraction

All data from databases were extracted into Microsoft Excel initially and then copied directly into a master sheet, which contained the included set of countries in one column. The lead author (VJ) checked the accuracy of the extraction process using the ‘IF’ command in Microsoft Excel to identify any values in the master sheet which did not match the relevant data from the database. If inaccuracies were identified they were rectified by manually overriding the inputted value with that in the original database file. A second author (TB) double-checked the accuracy of key data through using scatter plots on STATA to identify outliers. Where outliers were identified this prompted a further check of the master sheet against the relevant databases, to ensure that these values were genuine.

### Statistical analysis

We first made histograms of each variable. Due to high skew in communicable disease and COVID-19 deaths, we applied log-transformation and reported outcomes as log communicable disease deaths and log COVID-19 deaths per 100,000 population, respectively. We made scatter plots to visualise the relationship between variables and outcome measures.

We fitted univariable and multivariable linear regression models to investigate the relationship between JEE score and i) log COVID-19 deaths per 100,000 population and ii) log communicable disease deaths per 100,000 population, with statistical significance at a P value <0.05. We constructed three multivariable models to investigate the relationship between JEE scores and COVID-19 deaths (one each for deaths at 3, 6 and 12 months into the pandemic) and one to investigate the relationship between JEE scores and communicable disease deaths.

We considered confounders based on their potential relationship with disease control and deaths and statistical significance on univariable regression. For multivariable models investigating COVID-19 deaths at 12 months, we adjusted for 1) the proportion of the population aged ≥65, 2) health system access (measured by UHC service index), 3) health expenditure as a proportion of GDP, 4) COVID-19 testing capacity (measured by positivity rates), and 5) the strength of government restrictions one month prior to deaths (measured by the Stringency Index). For the multivariable models investigating COVID-19 deaths at 3 and 6 months we repeated this method but excluded test positivity rates, since data were missing for 43 countries, and instead included strength of democratic governance. National wealth (measured by Gross National Income per capita [10]) was excluded in the main analysis due to multicollinearity [21] with UHC service index (r = 0.75).

For the model investigating deaths from communicable diseases, potential confounders included 1) proportion of the population aged ≥65, 2) health system access, 3) national wealth, 4) strength of democratic governance, and 5) pre-pandemic international tourist arrivals.

Sensitivity analysis was performed to test the robustness of our findings (S3–S6 Tables), by adding originally excluded variables to each respective multivariable model and excluding test positivity rates from the model investigating COVID-19 deaths at 12 months, due to missing data for 32 countries. We used Microsoft Excel and Stata version 16 in the analysis.

### Ethics

Data used in this study were all open-access, obtained through routine data sources and collected at the population-level. There were no human or animal participants involved directly in this study and no ethical approval was required.

## Results

We identified and analyzed a total of 96 JEE reports. Data on COVID-19 deaths were unavailable for five countries (Cambodia, Eritrea, Federated States of Micronesia, Laos and Turkmenistan), leaving 91 for analysis (Table 1). High-income countries and the America and Europe region had the highest JEE scores, and low-income countries and the African region had the lowest scores. The median JEE score (as a percentage of the maximum possible score) across all countries was 50% (IQR 37.9–66.0%). Scores varied across JEE domains, with the highest scores for ‘detect’ (median 58.5, IQR 51.1–71.0), followed by ‘prevent’ (median 54.3, IQR 37.6–65.5) and ‘respond’ (median 43.7, IQR 30.6–66.4).

**Table 1:** Joint External Evaluation scores by country-income group and geographical region (N = 96).

		Number of countries	Median JEE score (IQR)
**Country-income group**	High-income	35	75.2 (57.8–88.9)
	Upper middle-income	27	48.7 (41.4–58.9)
	Lower middle-income	32	37.3 (34.0–46.2)
	Low-income	2	29.7 (29.0–30.5)
**Region**	Americas	2	90.1 (86.9–93.2)
	Europe	14	74.1 (58.0–84.7)
	Western Pacific	11	59.0 (52.9–92.1)
	Eastern Mediterranean	17	58.9 (50.1–75.2)
	South East Asia	8	47.8 (42.6–60.1)
	Africa	44	38.4 (34.2–45.5)

All of the investigated factors included in the multivariable model for COVID-19 deaths, were significantly predictive for deaths at all time points on univariable regression. Similarly, all of the factors included in the multivariable model investigating communicable disease deaths (2019) were significantly predictive in univariable models. Pre-pandemic tourism was not significantly associated with COVID-19 deaths on univariable regression, and was therefore excluded from multivariable models. The proportion of GDP spent on health was not significantly associated with communicable disease deaths on univariable regression and was therefore excluded from the multivariable model.

**Fig 1:**
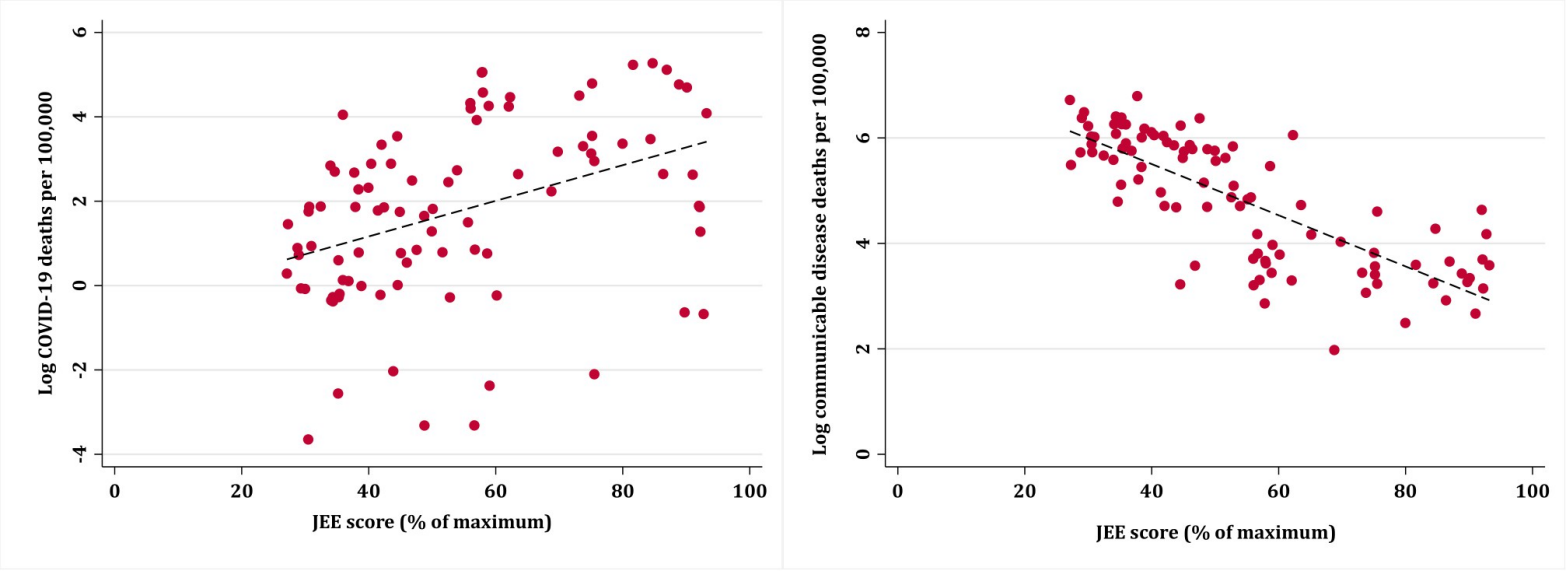
COVID-19 deaths (10^th^ March 2021) and communicable disease deaths (2019) by country Joint External Evaluation scores.

JEE score was positively associated with log COVID-19 deaths at 12 months into the pandemic but negatively associated with log communicable disease deaths (Fig 1). These relationships were statistically significant on univariable linear regression (S1 and S2 Tables). However, on multivariable linear regression (Table 2) the positive association between JEE scores and log COVID-19 deaths became non-significant at 6 and 12 months, while the negative association between JEE scores and log communicable disease deaths remained statistically significant (β 0.03, p = 0.003), with a 0.03 decrease in log communicable disease deaths for every percentage point increase in JEE score.

**Table 2:** Association between Joint External Evaluation scores and COVID-19 deaths per 100,000 population from multivariable linear regression models.

	Log COVID-19 deaths									Log communicable disease deaths (2019) ^d^		
	3 months^a^			6 months^b^			12 months^c^					
	Coefficient (95% CI)	P-value	R^2^	Coefficient (95% CI)	P-value	R^2^	Coefficient (95% CI)	P-value	R^2^	Coefficient (95% CI)	P-value	R^2^
**JEE score**	0.05 (0.009–0.09)	0.02	0.38	0.02 (-0.02–0.06)	0.27	0.29	0.03 (-0.01–0.07)	0.19	0.60	-0.03 (-0.04 –-0.01)	0.003	0.75

^a^ Adjusted for % population over 65 years, UHC index, % GDP spent on health, EIU Democracy Index, and OxCGRT Stringency Index from May 10^th^ 2020.

^b^ Adjusted for % population over 65 years, UHC index, % GDP spent on health, EIU Democracy Index, and OxCGRT Stringency Index from August 10^th^ 2020.

^c^ Adjusted for % population over 65 years, UHC index, % GDP spent on health, test positivity rate at 12 months, and OxCGRT Stringency Index from Feb 10^th^ 2021.

^d^ Adjusted for % population over 65 years, UHC index, GNI per capita, EIU Democracy Index, and international tourist arrivals (2019).

Abbreviations: EIU—Economist Intelligence Unit, GDP–Gross Domestic Product, GNI–Gross National Income, OxCGRT, Oxford COVID-19 Government Response Tracker, UHC–Universal Health Coverage.

Higher test positivity rates, representing poor testing capacity or high levels of transmission, were associated with higher log COVID-19 deaths at all time points. Stringency Index was also associated with higher log COVID-19 deaths at all time points but adding test positivity rates into the model at least partially attenuated this positive association (S3–S5 Tables). While UHC indices (β -0.04 p<0.001) and international tourist arrivals were associated with communicable disease deaths (β 0.02, p = 0.002), they were not significantly associated with COVID-19 deaths.

Review of scatter plots of residuals against fitted values showed no violations of heteroskedasticity and quantile plots of residuals showed no departures from normality in any of the models. Sensitivity analysis showed that these results remained valid even after altering the variables included in each multivariable model (S3–S6 Tables).

The proportion of the variation in COVID-19 deaths at 12 months and communicable disease deaths explained by both models was high, with R^2^ values of 0.60 and 0.75 respectively.

## Discussion

Countries with higher JEE scores were associated with significantly fewer overall communicable disease deaths, but not COVID-19 deaths. This finding remained even after accounting for a range of other factors that could influence the distribution of COVID-19 deaths across countries and after considering multiple time points. This suggests that the JEE may be better suited to assessing epidemics of known diseases compared to a pandemic of a novel pathogen. Test positivity rates were the only factor strongly associated with more COVID-19 deaths at 12 months, further supporting the notion that the determinants of the impact of epidemics and pandemics may differ.

Epidemics of known diseases and pandemics of novel pathogens pose greatly different challenges when it comes to testing. The JEE explicitly assesses testing for infectious diseases through the availability of diagnostics, laboratory systems and real-time indicator and event-based surveillance systems [22]. It does not focus on scale, size and surge capacity of national infectious disease testing systems. Developing and scaling up the availability of tests, including RT-PCR and rapid tests such as the lateral flow assay, has been an unprecedented challenge for all countries during the pandemic [23]. Although the first diagnostic tests for COVID-19 were developed within 2 weeks of the reference genome being published [24], countries scrambled to compete for scarce global resources to expand testing programmes, leading to vast global inequities in access to testing. For example, in April 2020, while the USA was approaching 4 million tests conducted, Nigeria, Africa’s most populous country with a population almost two-thirds that of the USA, was nearing only 7000 tests [25]. In our multivariable analysis, even after accounting for JEE scores, weaker testing capacity (indicated by high test positivity rates) was significantly associated with increased COVID-19 deaths at 12 months. Given the existing but limited assessment of infectious disease testing capacities and scalability in the current JEE, this may be an area for early reform, if the JEE is to be used as an effective pandemic preparedness risk assessment tool.

Although the JEE is a self-assessment tool and was not designed to compare countries’ performance, the indicators should reflect necessary components of epidemic preparedness; logically, countries with higher scores should be better prepared to respond to a pandemic and limit transmission and mortality. The fact that JEE scores were not associated with fewer COVID-19 deaths goes against this expectation, but is in line with previous findings [4,5]. Possible reasons for the discrepancy in associations between JEE scores and COVID-19 deaths compared to other communicable disease deaths include 1) For many common infections, the natural history of disease is well understood, clinical tests are available and surveillance systems are well-established. In the early stages of the COVID-19 pandemic, global testing capacity was inadequate, including in many high-income country global travel hubs, which made countries vulnerable to imported cases. Consequently, the true burden of COVID-19 cases in real-time was likely underestimated [26], limiting effective public health action; 2) The pandemic required outbreak response capacities of a scale not seen for smaller epidemics of known diseases. Existing infrastructure for testing, contact tracing, infection control and clinical management had to be rapidly scaled up and reorganized [27,28]; 3) In the absence of vaccines or therapeutics, few disease control measures for COVID-19 were initially available, as they might be for other common epidemic-prone diseases, such as tuberculosis or measles. In this context, individual behaviours across populations, including physical distancing and mask wearing, became an important determinant of spread [29], in turn affected by public risk communication as well as individual attitudes toward infectious diseases, personal risk and freedoms; 4) Unlike for small outbreaks of known diseases, decision-making on control measures took place largely outside of traditional public health agencies, with political leaders having to weigh trade-offs in a highly public time-pressured environment and with significant scientific uncertainty; and 5) In its current form, the JEE considers a range of factors but focuses on the existence of capacities, such as policies, procedures, and systems, rather than the quality of them, which is more difficult to demonstrate, and the ability to deliver these capacities at the speed and scale required in a pandemic. Other limitations of the JEE, such as potential bias created from self-assessment and limited scope [3] may further help explain the disparity in the relationship between communicable disease and COVID-19 deaths.

Limitations of our study include: 1) Our study may be vulnerable to selection bias given that not all countries have volunteered for the JEE (analysable data were available only from 91 countries, less than half of the world’s total), and those that have may be different to those that have not; 2) The JEE was not designed as a comparative tool across settings but rather to allow countries to identify areas of weakness and track progress. JEE implementation and interpretation may therefore vary across different countries and over time, independent of other factors; 3) We used COVID-19 deaths in our analysis, which may not be recorded in the same way across countries, although these data are likely less susceptible to biases related to testing and healthcare-seeking compared to some other indicators, such as numbers of cases; 4) Underreporting of deaths due to inadequate mortality surveillance, if associated with lower JEE scores, could have biased the results. We considered open-access data on excess mortality [30] as an alternative and more comprehensive way of measuring COVID-19 impact but, due to large amounts of missing and poor data quality, did not pursue this approach; and 5) Our study covered only a 12-month period during which vaccine distribution has been highly unequal across the world [31]. The associations between JEE scores and COVID-19 deaths may change as the pandemic progresses.

Our findings have implications for global health policy. Many of the countries with the highest numbers of cases and deaths from COVID-19 are also considered to have the most robust IHR capacities and health systems as measured by the JEE. The IHR, and the JEE that is derived from it, may therefore require design change or supplementation through other mechanisms if the wide range of factors involved in preventing, detecting and responding to both future pandemics and epidemics of known diseases are to be comprehensively captured through country risk assessments. It may be necessary to develop and test more complex and demanding requirements within tools like the JEE, simulation exercises and after-action reviews, to improve future emergency preparedness by better measuring real-world capabilities. For example, approaches could better account for the altered response capacities, disease control measures, and political considerations that may be necessary in dealing with pandemics and novel pathogens. Our results also underscore the importance of testing capacity, which may prove a sensible early target for the pandemic-focussed reform of existing tools.

Given that the existing public health response capacities outlined in Annex 1 of the IHR [32] are still not adequately implemented in many countries, including more requirements within the JEE may not translate into better emergency preparedness. Indeed, one critical implication from our findings is the need to recognize the limitations in the utility of JEE as a measure of pandemic preparedness, given the uncertainty of how any future pandemic may look and the potentially devastating consequences of complacency or overconfidence in response capabilities. Various factors may improve the implementation of IHR and deserve attention alongside the scrutiny of risk assessment tools. These include stronger public health systems, workforce and institutions; financial incentives; appropriate decentralisation of powers; community and private sector engagement; upskilling the public health workforce; improved data collection, transparency and sharing; supportive legal instruments; national leadership; and regional collaboration [33,34].

Much of the recent discourse on global health security has emphasised integration with the One Health, UHC and health system strengthening agendas [35–37], underscoring the links between humans, animals, ecosystems and health systems. The discussion on pandemic preparedness must expand to consider essential public health functions [38], providing an opportunity to consider pandemic risk and preparedness in the broader context of actions required to promote and protect health. High quality test, trace and isolate systems (often operating outside the traditional structures of health systems) have proved an essential part of early pandemic response in many countries [39]. Nor can the focus be entirely on the implicated pandemic pathogen; during the first peak in the United Kingdom (UK), 96% of COVID-19 deaths were in individuals with at least one pre-existing medical condition, most of which are chronic non-communicable diseases [40]. Those living in more densely populated and deprived areas, working in high-risk occupations with poor access to healthcare, are at a high risk of infection and severe illness [41,42]. Sociocultural and political factors have also been found to be important predictors of COVID-19 deaths across countries [12,43,44] compared to smaller-scale outbreaks of known infectious diseases. Capturing such vulnerabilities as part of assessments of preparedness will require more research and an expansion in ambition, but may help to improve the utility of risk assessments. Effective systems of public sector governance will be required to operationalize essential public health functions. Pandemic preparedness assessments must also build an understanding of how evidence is used in decision-making, and how well-informed, fair, and inclusive strategic decisions are likely to be in emergency scenarios, regardless of more specific public health vulnerabilities or capacities.

## Supporting information

S1 Table Univariable linear regression for log COVID-19 deaths. (DOCX)

S2 Table Univariable linear regression for log communicable disease deaths (2019). (DOCX)

S3 Table Multivariable linear regression models: The association between JEE score and log COVID-19 deaths at 3 months. (DOCX)

S4 Table Multivariable linear regression models: The association between JEE score and log COVID-19 deaths at 6 months. (DOCX)

S5 Table Multivariable linear regression models: The association between JEE score and log COVID-19 deaths at 12 months. (DOCX)

S6 Table Multivariable linear regression models: The association between JEE score and log communicable disease deaths (2019). (DOCX)

S1 Data JEE deaths project raw data. (XLSX)
