# Supplementary material for: Joint External Evaluation scores and communicable disease deaths: An ecological study on the difference between epidemics and pandemics
Source: PLOS Glob Public Health. 2022 Aug 11;2(8):e0000246. doi: 10.1371/journal.pgph.0000246 (PMC10021717; doi:10.1371/journal.pgph.0000246)
Supplement: S1 Table — (DOCX) [file pgph.0000246.s001.docx]

**S1 Table**

Table 1 – Univariable linear regression for log COVID-19 deaths

|  | **Deaths at 3 months** | | **Deaths at 6 months** | | **Deaths at 12 months** | |
| --- | --- | --- | --- | --- | --- | --- |
| Factor | Coefficient (95% CI) | P-value | Coefficient (95% CI) | P-value | Coefficient (95% CI) | P-value |
| JEE score | 0.05 (0.03 – 0.07) | 0.001 | 0.04 (0.02 – 0.05) | <0.001 | 0.04 (0.02 – 0.06) | <0.001 |
| % population ≥ 65 years | 0.16 (0.09 – 0.22) | <0.001 | 0.08 (0.02 – 0.14) | 0.01 | 0.14 (0.07 – 0.21) | <0.001 |
| UHC Index | 0.05 (0.03 – 0.08) | <0.001 | 0.04 (0.02- 0.07) | <0.001 | 0.06 (0.04 – 0.08) | <0.001 |
| GNI per capita | -0.00005 (-0.00004 - -0.00007) | <0.001 | -0.00003 (0.00002 - -0.00005) | <0.001 | 0.00003 (0.00001 – 0.00005) | 0.001 |
| GDP (%) spent on health | 0.20 (0.04 – 0.35) | 0.02 | 0.15 (0.02 – 0.29) | 0.03 | 0.24 (0.09 – 0.39) | 0.002 |
| EIU Democracy Index | 0.34 (0.13 – 0.55) | 0.002 | 0.22 (0.03 – 0.41) | 0.03 | 0.26 (0.04 – 0.48) | 0.02 |
| International tourist arrivals (2019) | 0.05 (0.001 – 0.10) | 0.02 | 0.03 (-0.008 – 0.07) | 0.12 | 0.29 (-0.01 – 0.07) | 0.18 |
| OxCGRT Stringency Index (2 months) | 0.04 (0.004 – 0.07) | 0.03 | - | - | - | - |
| OxCGRT Stringency Index (5 months) | - | - | 0.03 (0.009 – 0.05) | 0.006 | - | - |
| OxCGRT Stringency Index (11 months) | - | - | - | - | 0.05 (0.03 – 0.07) | <0.001 |
| Test positivity rate (3 months) | 0.12 (0.05 – 0.20) | 0.002 | - | - | - | - |
| Test positivity rate (6 months) | - | - | 0.06 (-0.005 – 0.13) | 0.07 | - | - |
| Test positivity rate (12 months) | - | - | - | - | 0.19 (0.12 – 0.26) | <0.001 |
