# Supplementary material for: Joint External Evaluation scores and communicable disease deaths: An ecological study on the difference between epidemics and pandemics
Source: PLOS Glob Public Health. 2022 Aug 11;2(8):e0000246. doi: 10.1371/journal.pgph.0000246 (PMC10021717; doi:10.1371/journal.pgph.0000246)
Supplement: S2 Table — (DOCX) [file pgph.0000246.s002.docx]

**S2 Table**

S2 Table – Univariable linear regression for log communicable disease deaths (2019)

| **Factor** | **Coefficient (95% CI)** | **P-value** |
| --- | --- | --- |
| JEE score | -0.05 (-0.06 - -0.04) | <0.001 |
| % population ≥ 65 years | -0.11 (-0.15 - -0.08) | <0.001 |
| UHC Index | -0.06 (-0.07 - -0.05) | <0.001 |
| GNI per capita | -0.00004 (-0.00005 - -0.00003) | <0.001 |
| GDP (%) spent on health | -0.06 (-0.15 – 0.02) | 0.15 |
| EIU Democracy Index | -0.19 (-0.31 - -0.07) | 0.002 |
| International tourist arrivals (2019) | -0.03 (-0.06 - -0.01) | 0.008 |
