# Supplementary material for: Joint External Evaluation scores and communicable disease deaths: An ecological study on the difference between epidemics and pandemics
Source: PLOS Glob Public Health. 2022 Aug 11;2(8):e0000246. doi: 10.1371/journal.pgph.0000246 (PMC10021717; doi:10.1371/journal.pgph.0000246)
Supplement: S3 Table — (DOCX) [file pgph.0000246.s003.docx]

**S3 Table**

Table 3 - Multivariable linear regression models: the association between JEE score and log COVID-19 deaths at 3 months

| Model | Variables included in model | Coefficient (95% CI) | P-value | R^2^ |
| --- | --- | --- | --- | --- |
| Main model | JEE score | 0.05 (0.009 – 0.09) | 0.02 | 0.39 |
|  | % population ≥ 65 years | 0.08 (-0.03 – 0.19) | 0.14 |  |
|  | UHC index | -0.03 (-0.08 – 0.03) | 0.32 |  |
|  | % GDP spent on health | 0.07 (-0.08 – 0.23) | 0.36 |  |
|  | EIU democracy index | 0.04 (-0.25 – 0.32) | 0.79 |  |
|  | Ox CGRT Stringency Index (May 10^th^ 2020) | 0.04 (0.02 – 0.07) | 0.003 |  |
| Main model plus test positivity rate | JEE score | 0.07 (0.009 – 0.13) | 0.03 | 0.59 |
|  | % population ≥ 65 years | 0.03 (-0.10 – 0.17) | 0.61 |  |
|  | UHC index | -0.02 (-0.10 – 0.06) | 0.64 |  |
|  | % GDP spent on health | 0.09 (-0.15 - 0.32) | 0.46 |  |
|  | EIU democracy index | 0.15 (-0.26 – 0.55) | 0.46 |  |
|  | Ox CGRT Stringency Index (May 10^th^ 2020) | 0.05 (0.005 – 0.09) | 0.03 |  |
|  | Test positivity rate (June 10th 2020) | 0.09 (0.03 – 0.16) | 0.006 |  |
| Main model plus GNI per capita | JEE score | 0.01 (-0.03 – 0.06) | 0.55 | 0.49 |
|  | % population ≥ 65 years | 0.07 (-0.03 – 0.17) | 0.17 |  |
|  | UHC index | -0.04 (-0.09 – 0.06) | 0.08 |  |
|  | % GDP spent on health | 0.08 (-0.07 – 0.22) | 0.30 |  |
|  | EIU Democracy Index | 0.12 (-0.15 – 0.38) | 0.39 |  |
|  | OxCGRT Stringency Index (May 10th 2020) | 0.05 (0.02 – 0.08) | 0.001 |  |
|  | GNI per capita | 0.00005 (0.000002 – 0.00008) | <0.001 |  |
| Main model plus international tourist arrivals | JEE score | 0.04 (-0.002 – 0.09) | 0.06 | 0.41 |
|  | % population ≥ 65 years | 0.10 (-0.02 – 0.21) | 0.10 |  |
|  | UHC index | -0.01 (-0.07 – 0.04) | 0.60 |  |
|  | % GDP spent on health | 0.06 (-0.11 – 0.23) | 0.46 |  |
|  | EIU Democracy Index | 0.005 (-0.30 – 0.31) | 0.97 |  |
|  | OxCGRT Stringency Index (May 10th 2020) | 0.05 (0.02 – 0.08) | 0.003 |  |
|  | International tourist arrivals (2019) | 0.004 (-0.04 – 0.05) | 0.86 |  |
