# Supplementary material for: Joint External Evaluation scores and communicable disease deaths: An ecological study on the difference between epidemics and pandemics
Source: PLOS Glob Public Health. 2022 Aug 11;2(8):e0000246. doi: 10.1371/journal.pgph.0000246 (PMC10021717; doi:10.1371/journal.pgph.0000246)
Supplement: S4 Table — (DOCX) [file pgph.0000246.s004.docx]

**S4 Table**

Table 4 - Multivariable linear regression models: the association between JEE score and log COVID-19 deaths at 6 months

| Model | Variables included in model | Coefficient (95% CI) | P-value | R^2^ |
| --- | --- | --- | --- | --- |
| Main model | JEE score | 0.02 (-0.02 – 0.06) | 0.27 | 0.29 |
|  | % population ≥ 65 years | 0.008 (-0.10 – 0.11) | 0.89 |  |
|  | UHC index | 0.002 (-0.05 – 0.05) | 0.92 |  |
|  | % GDP spent on health | 0.08 (-0.07 – 0.22) | 0.30 |  |
|  | EIU democracy index | 0.14 (-0.13 – 0.41) | 0.30 |  |
|  | Ox CGRT Stringency Index (August 10^th^ 2020) | 0.04 (0.02 – 0.06) | 0.001 |  |
| Main model plus test positivity rate | JEE score | 0.07 (0.005 – 0.13) | 0.04 | 0.35 |
|  | % population ≥ 65 years | -0.05 (-0.19 – 0.09) | 0.47 |  |
|  | UHC index | -0.03 (-0.10 – 0.05) | 0.48 |  |
|  | % GDP spent on health | 0.18 (-0.05 - 0.41) | 0.12 |  |
|  | EIU democracy index | 0.09 (-0.32 – 0.50) | 0.66 |  |
|  | Ox CGRT Stringency Index (August 10^th^ 2020) | 0.02 (-0.02 – 0.05) | 0.28 |  |
|  | Test positivity rate (September 10th 2020) | 0.09 (0.02 – 0.15) | 0.02 |  |
| Main model plus GNI per capita | JEE score | -0.001 (0.04 – 0.04) | 0.95 | 0.34 |
|  | % population ≥ 65 years | 0.004 (-0.10 – 0.11) | 0.93 |  |
|  | UHC index | -0.008 (-0.06 – 0.04) | 0.73 |  |
|  | % GDP spent on health | 0.08 (-0.06 - 0.22) | 0.29 |  |
|  | EIU Democracy Index | 0.19 (-0.07 – 0.46) | 0.16 |  |
|  | OxCGRT Stringency Index (August 10th 2020) | 0.04 (0.02 – 0.06) | <0.001 |  |
|  | GNI per capita | 0.09 (0.000004 – 0.00006) | 0.03 |  |
| Main model plus international tourist arrivals | JEE score | 0.02 (-0.02 – 0.06) | 0.27 | 0.33 |
|  | % population ≥ 65 years | 0.02 (-0.09 – 0.13) | 0.76 |  |
|  | UHC index | 0.007 (-0.04 – 0.06) | 0.79 |  |
|  | % GDP spent on health | 0.09 (-0.07 - 0.25) | 0.27 |  |
|  | EIU Democracy Index | 0.14 (-0.14– 0.43) | 0.32 |  |
|  | OxCGRT Stringency Index (August 10th 2020) | 0.04 (0.02 – 0.06) | 0.001 |  |
|  | International tourist arrivals (2019) | -0.02 (-0.06 – 0.02) | 0.41 |  |
