# Supplementary material for: Joint External Evaluation scores and communicable disease deaths: An ecological study on the difference between epidemics and pandemics
Source: PLOS Glob Public Health. 2022 Aug 11;2(8):e0000246. doi: 10.1371/journal.pgph.0000246 (PMC10021717; doi:10.1371/journal.pgph.0000246)
Supplement: S5 Table — (DOCX) [file pgph.0000246.s005.docx]

**S5 Table**

Table 5 - Multivariable linear regression models: the association between JEE score and log COVID-19 deaths at 12 months

| Model | Variables included in model | Coefficient (95% CI) | P-value | R^2^ |
| --- | --- | --- | --- | --- |
| Main model | JEE score | 0.03 (-0.01 – 0.07) | 0.19 | 0.60 |
|  | % population ≥ 65 years | 0.01 (-0.07 – 0.10) | 0.78 |  |
|  | UHC index | 0.008 (-0.05 – 0.06) | 0.78 |  |
|  | % GDP spent on health | 0.15 (-0.30 – 0.33) | 0.10 |  |
|  | Test positivity rate (March 10^th^ 2021) | 0.19 (0.13 – 0.25) | <0.001 |  |
|  | OxCGRT Stringency Index (Feb 10^th^ 2021) | 0.02 (-0.003 – 0.05) | 0.08 |  |
| Main model without test positivity rate | JEE score | 0.02 (-0.03 – 0.06) | 0.48 | 0.36 |
|  | % population ≥ 65 years | 0.03 (-0.06 – 0.13) | 0.46 |  |
|  | UHC index | 0.008 (-0.05 – 0.06) | 0.77 |  |
|  | % GDP spent on health | 0.11 (-0.04 - 0.27) | 0.14 |  |
|  | Ox CGRT Stringency Index (Feb 10^th^ 2021) | 0.04 (0.02 – 0.07) | <0.001 |  |
| Main model plus EIU Democracy Index | JEE score | 0.03 (-0.01 – 0.07) | 0.15 | 0.63 |
|  | % population ≥ 65 years | 0.07 (-0.04 – 0.17) | 0.20 |  |
|  | UHC index | -0.002 (-0.06 - 0.06) | 0.93 |  |
|  | % GDP spent on health | 0.19 (0.01 – 0.37) | 0.04 |  |
|  | Test positivity rate (March 10^th^ 2021) | 0.19 (0.12 – 0.25) | <0.001 |  |
|  | OxCGRT Stringency Index (Feb 10^th^ 2021) | 0.02 (-0.005 – 0.05) | 0.10 |  |
|  | EIU Democracy Index | -0.26 (-0.57 – 0.04) | 0.09 |  |
| Main model plus GNI per capita | JEE score | 0.02 (-0.03 – 0.07) | 0.54 | 0.61 |
|  | % population ≥ 65 years | 0.02 (-0.07 – 0.10) | 0.69 |  |
|  | UHC index | 0.004 (-0.05 - 0.06) | 0.88 |  |
|  | % GDP spent on health | 0.15 (-0.03 – 0.33) | 0.07 |  |
|  | Test positivity rate (March 10^th^ 2021) | 0.19 (0.13 – 0.25) | <0.001 |  |
|  | OxCGRT Stringency Index (Feb 10^th^ 2021) | 0.02 (-0.002 – 0.05) | 0.07 |  |
|  | GNI per capita | 0.00001 (-0.00001 – 0.0004) | 0.29 |  |
| Main model plus international tourist arrivals | JEE score | 0.03 (-0.01 – 0.08) | 0.16 | 0.60 |
|  | % population ≥ 65 years | -0.00008 (-0.09 – 0.09) | 0.99 |  |
|  | UHC index | 0.009 (-0.05 - 0.07) | 0.78 |  |
|  | % GDP spent on health | 0.21 (0.01 – 0.40) | 0.04 |  |
|  | Test positivity rate (March 10^th^ 2021) | 0.18 (0.11 – 0.24) | <0.001 |  |
|  | OxCGRT Stringency Index (Feb 10^th^ 2021) | 0.02 (-0.006 – 0.05) | 0.11 |  |
|  | International tourist arrivals (2019) | -0.02 (-0.06 – 0.02) | 0.24 |  |
