# Supplementary material for: Joint External Evaluation scores and communicable disease deaths: An ecological study on the difference between epidemics and pandemics
Source: PLOS Glob Public Health. 2022 Aug 11;2(8):e0000246. doi: 10.1371/journal.pgph.0000246 (PMC10021717; doi:10.1371/journal.pgph.0000246)
Supplement: S6 Table — (DOCX) [file pgph.0000246.s006.docx]

**S6 Table**

S6 Table - Multivariable linear regression models: the association between JEE score and log communicable disease deaths (2019)

| Model | Variables included in model | Coefficient (95% CI) | P-value | R^2^ |
| --- | --- | --- | --- | --- |
| Main model | JEE score | -0.03 (-0.04 – -0.009) | 0.003 | 0.75 |
|  | % population ≥ 65 years | -0.01 (-0.05 – 0.03) | 0.51 |  |
|  | UHC index | -0.04 (-0.06 – -0.02) | <0.001 |  |
|  | GNI per capita | 0.000007 (-0.00002 – 0.000004) | 0.21 |  |
|  | EIU Democracy Index | 0.10 (-0.002 – 0.21) | 0.06 |  |
|  | International tourist arrivals (2019) | 0.02 (0.009 – 0.04) | 0.002 |  |
| Main model plus % GDP spent on health | JEE score | -0.03 (-0.04 – -0.009) | 0.002 | 0.76 |
|  | % population ≥ 65 years | -0.02 (-0.06 – 0.18) | 0.27 |  |
|  | UHC index | -0.04 (-0.06 – -0.02) | <0.001 |  |
|  | GNI per capita | 0.000006 (-0.00002 – 0.000004) | 0.23 |  |
|  | EIU Democracy Index | 0.09 (-0.01– 0.19) | 0.09 |  |
|  | International tourist arrivals (2019) | 0.02 (0.004 - -0.04) | 0.01 |  |
|  | % GDP spent on health | 0.06 (0.003 – 0.12) | 0.04 |  |
